# Hybrids between common and Antarctic minke whales are fertile and can back-cross

**DOI:** 10.1186/1471-2156-14-25

**Published:** 2013-04-15

**Authors:** Kevin A Glover, Naohisa Kanda, Tore Haug, Luis A Pastene, Nils Øien, Bjørghild B Seliussen, Anne G E Sørvik, Hans J Skaug

**Affiliations:** 1 Institute of Marine Research, PO box 1870, Nordnes N-5817, Bergen, Norway; 2 Institute of Cetacean Research, Toyomi-cho 4-5, Chuo-ku, Tokyo 104-0055, Japan; 3 Institute of Marine Research, PO box 6404, Tromsø N-9294, Norway; 4 Department of Mathematics, University of Bergen, Bergen, N-5008, Norway

## Abstract

**Background:**

Minke whales are separated into two genetically distinct species: the Antarctic minke whale found in the southern hemisphere, and the common minke whale which is cosmopolitan. The common minke whale is further divided into three allopatric sub-species found in the North Pacific, southern hemisphere, and the North Atlantic. Here, we aimed to identify the genetic ancestry of a pregnant female minke whale captured in the North Atlantic in 2010, and her fetus, using data from the mtDNA control region, 11 microsatellite loci and a sex determining marker.

**Results:**

All statistical parameters demonstrated that the mother was a hybrid displaying maternal and paternal contribution from North Atlantic common and Antarctic minke whales respectively. Her female fetus displayed greater genetic similarity to North Atlantic common minke whales than herself, strongly suggesting that the hybrid mother had paired with a North Atlantic common minke whale.

**Conclusion:**

This study clearly demonstrates, for the first time, that hybrids between minke whale species may be fertile, and that they can back-cross. Whether contact between these species represents a contemporary event linked with documented recent changes in the Antarctic ecosystem, or has occurred at a low frequency over many years, remains open.

## Background

While the number of minke whale species and sub-species is still a matter of discussion [1], based upon morphological [2] and genetic data [3,4], minke whales are presently considered as two species: the Antarctic minke (*Balaenoptera bonaerensis*) thought to be present in the southern hemisphere, and the common minke (*B. acutorostrata*) which is cosmopolitan. The common minke whale is further divided into three allopatric sub-species, found in the North Atlantic (*B. acutorostrata acutorostrata*), the North Pacific (*B. a. scammoni*) and in the southern oceans (dwarf common minke whale: *B.a.* unnamed sub-species) [5]. Analyses of mtDNA data indicate that the two species may have established from a separation in the southern hemisphere approximately 5 million years ago [4]. Furthermore, mtDNA data suggests that the three sub species for *B. acutorostrata* were established approximately 1.5 million years ago. Looking at microsatellite DNA markers, large and in some markers multiple fixed allele differences can be observed between these species [6]. Thus, these markers provide an opportunity to perform species identification.

A recent genetics study documented, for the first time, the presence of a *B. bonaerensis* north of the Arctic circle [6]. Prior to that study, *B. bonaerensis* was considered to exist exclusively in the southern hemisphere or sub-tropical regions, and did not overlap in both time and space with *B. a. acutorostrata* that is thought to exist exclusively in the North Atlantic. All species of minke whales probably use tropical and sub-tropical regions for overwintering. However, a combination of the fact that there are differences in timing of the seasons between northern and southern hemispheres, and that the whales display synchronized parallel seasonal migrations between poles and equatorial regions, are likely to reduce the probability of these species overlapping in both time and space. In addition to observing an Antarctic minke in the Arctic, a second whale deviating from the standard genetic pattern of *B. a. acutorostrata* in the Atlantic has been reported in the northern Atlantic [6]. This individual is the first documented hybrid between minke whale species, and based upon an analysis of both nuclear DNA (inherited from both parents) and mtDNA (maternally inherited), was demonstrated to consist of maternal contribution from *B. bonaerensis* and most likely paternal contribution from *B. a. acutorostrata*. Documented examples of interspecific hybrids in *Balaenopteridae* whales are very rare, although this has previously been reported between blue (*B. musculus*) and fin (*B. physalus*) whales [7-9].

**Figure 1:**
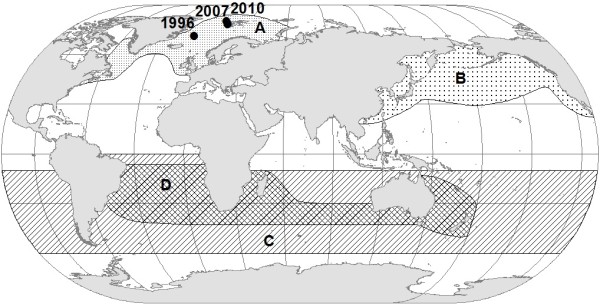
**Map of the distribution of the different minke whale species and sub-species, including locations of capture for pregnant minke whale suspected to be a hybrid in 2010. A**: *Balaenoptera a. acutorostrata*, **B**: *B. a. scammoni*, **C**: *B. bonaerensis*, **D**: *B. a. unnamed subspecies* (dwarfs).

Norway conducts a commercial harvest of *B. a. acutorostrata* in the Northeast Atlantic, and each year, approximately 500 whales are captured. In order to enforce domestic regulation and compliance within this harvest, an individual-based DNA register (hereon referred to as the NMDR) has been maintained since 1996 [10]. This register contains individual genetic profiles for almost all *B. a. acutorostrata* harvested by Norwegian whalers in the period 1996-present. In the harvest year 2010, a female whale, captured north of the Arctic circle, position 79°45′N 9°32′E on 1 July (Figure 1), deviated from the standard genetic profile for *B. a. acutorostrata* in the Northeast Atlantic. This individual, suspected to be a hybrid based upon preliminary inspection of the genetic data, was pregnant. Here, we present identification of this suspected hybrid, and her fetus, using a mixture of mtDNA and microsatellite genetic markers.

## Methods

### Genotyping and the data set

The genetic analyses upon which the NMDR is based are currently run at the molecular genetics laboratory at the Institute of Marine Research (IMR) in Bergen, Norway. The analyses consist of sequencing part of the mtDNA control region, in addition to analysis of 10 microsatellite DNA markers and a fragment based sex determining marker. These analyses, including full description of markers, amplifications conditions and reagents [10], and detailed analysis of genotyping consistency [11] have been described previously.

Samples from the suspected hybrid and her fetus, were subject to analysis with the standard set of genetic markers implemented within the NMDR. However, not all of these genetic markers provide reliable genotypes in the other minke whale species and sub-species [6]. Thus, genetic data from only eight of the standard markers used in the NMDR were used for the identification analyses in the present study (see results). In addition to these eight markers, an extra set of three microsatellite markers, which provide very strong (species-diagnostic in many instances) genetic differentiation among minke whale species and sub-species [6] were analysed for these two whales. Thus, the identifications in the present study are based upon 11 microsatellite markers.

In addition to samples from the suspected hybrid and her fetus, 9 dwarf minke whales (*B.a.* unnamed sub-species – hereon referred to as “dwarfs”) were analysed for the 11 microsatellite markers. These samples were not sent to IMR for analysis, but were analysed at the Institute of Cetacean Research (ICR) in Japan. Inter-laboratory calibration for microsatellite markers is notoriously challenging due to the fact that the alleles are scored in a polymer matrix in relation to DNA fragments of known size (i.e., the size-standard), a process influenced by local instrument chemistry. Nevertheless, calibration of the standard set of microsatellite markers implemented in the NMDR has been previously achieved between four separate laboratories that have been involved with genotyping the samples upon which the register is based [11], and has for example also been achieved in a European project for Atlantic salmon (*Salmo salar* L.) data coming from multiple laboratories [12]. In order to calibrate microsatellite analyses between the laboratories at ICR and IMR, samples have been exchanged as part of a previous study [6]. Fifteen of these samples (5× *B. bonaerensis*, 5× *B. a. acutorostrata*, and 5× *B. a. scammoni*) were genotyped at ICR for the microsatellites implemented in this study. This permitted the calibration of the scoring systems between these two laboratories, and thus permitted transformation of the genotyping score for the 9 dwarfs genotyped at ICR, into the scoring system of IMR (Additional file 1: Table S1). Thus, in addition to the suspected hybrid and her fetus, 9 dwarf minke whales were also genotyped specifically for this study. All other samples used here originate from a previous study [6].

The primary genetic baseline, i.e., the set of reference samples used to compare the suspected hybrid and her fetus’ microsatellite DNA profiles to for identification, was obtained from the 9 newly genotyped dwarfs, 91 *B. bonaerensis*, 91 *B. a. acutorostrata*, and 95 *B. a. scammoni*. The dwarfs were not included in the “genetic baseline” for all statistical identification computations. This is due to the fact that some of the statistical identification methods (see below) require reliable estimates of each species’ allele frequencies. The inclusion or exclusion of the dwarfs from each of the specific analyses has been indicated in the relevant sections in the results.

In addition to the primary genetic baseline to identify the suspected hybrid and her fetus, the first documented inter-oceanic migrating *B. bonaerensis* (1996 whale), and first observed hybrid between *B. bonaerensis* and *B. a. acutorostrata* (2007 whale) [6] were included in some of the genetic comparisons. The latter individual was specifically included in these analyses in order to attempt to conclusively resolve paternity for this individual. This is because the dwarfs were not available for the previous study and thus paternity was not conclusively resolved. The results from this re-analysis of the hybrid whale captured in 2007 are addressed in their own results section for clarity.

All of these samples, including those from the Norwegian minke whale DNA register and the Japanese whale research programs under special permit in both the western North Pacific and Antarctic, existed prior to this study.

### Statistical analyses

MtDNA sequences from the suspected hybrid and her fetus were aligned to sequences of *B. bonaerensis* and sub-species of *B. acutorostrata*[13]. The genealogy of the mtDNA haplotypes was estimated using the Neighbor-Joining method [14] as implemented in the program PHYLIP. Genetic distances among haplotypes were estimated using the program DNADIST of PHYLIP, based on Kimura-2-parameter model. A transition-transversion ratio of 5:1 was used (ratios between 2:1 and 20:1 were tested and had no detectable effect on the clustering). The genealogy was rooted using the homologous sequence from nine baleen whale species [15]. To estimate support for each node, a total of 1,000 bootstrap simulations were conducted and the majority-rule consensus genealogy estimated.

Summary statistics for the 11 microsatellite markers for the four species and sub-species were computed using the programs MSA [16], and Genepop [17] (using the newer web-based version for Genepop). F_ST_ estimations both globally and pair-wise among the species and sub-species were computed in MSA which implements the W&C estimator [18]. Identification of the suspected hybrid and her fetus was conducted using several statistical approaches. While some of the principles of the statistical tests implemented here overlap with each other, they use different analytical approaches and thus complement each other.

Bayesian cluster analysis was performed using the program Structure [19,20]. Bayesian cluster analysis represents a powerful tool with which to investigate the genetic relationship among individuals and groups of individuals of potentially mixed and admixed origin. The program assigns individuals to genetic clusters/populations without taking any prior information regarding each individual into consideration, i.e., individual whales are not organized into pre-determined populations or species. In this program, an admixture model, no population prior for all individuals, and the burn-in set to 500 000 MCMC steps, followed by a further 500 000 steps was used. This was conducted for numbers of genetic groups/populations (*K*) set from 1–6, each with 3 iterations. All samples detailed in Table 1 were run in this program.

**Table 1:** List of samples included in the present study

**Sample**	**N**	**Genotyped at IMR?**	**Purpose of sample**	**Included in previous study [**[6]**]?**
*B. bonaerensis*	91	Yes	Included in genetic baseline	Yes
*B. a. acutorostrata*	91	Yes	Included in genetic baseline	Yes
*B. a. scammoni*	95	Yes	Included in genetic baseline	Yes
*B. bonaerensis* migrant, and *B. bonaerensis* x *B. a.* hybrid	2	Yes	Used for some comparisons	Yes
*B. a.* “dwarfs” (unnamed sub species)	9	No – calibrated	Included in genetic baseline for some analyses	No
Pregnant suspected hybrid whale and her female fetus	2	Yes	Main samples to be identified	No

In addition to Bayesian cluster analysis, genetic assignment was conducted in the program GeneClass2 [21], using a genetic baseline consisting of the three minke whale species and sub-species which had large sample sizes (i.e., excluding the dwarfs), in addition to three sets of hybrids produced in the program HYBRIDLAB1.0 [22]. These F1 hybrids were simulated between *B. a. acutorostrata* and *B. bonaerensis*, and *B. a. scammoni* and *B. bonaerensis*, and finally, *B. a. acutorostrata* and *B. a. scammoni*. The baseline for the genetic assignment tests did not include the dwarfs as this sample did not include enough individuals from which to produce a reliable estimate of the allele frequencies for this sub-species with which to conduct assignment. The assignment power of this data set was tested via self-assignment using the leave one out approach. Genetic assignment of the suspected hybrid and her fetus was first conducted using the direct assignment approach. This places the unknown individual(s) in question into the genetically most similar baseline sample. This classification is conducted irrespective of absolute level of similarity. In data sets where one or more of the potential baseline samples (populations or species) is not present, it is also important to get a statistical measurement of the level of similarity between the unknown individual(s) and each of the baseline samples. In order to achieve this, the probability of being able to exclude the genotype of the unknown individual(s) from each of the genetic profiles for the baseline samples (in this case species and hybrids) is computed. Exclusion was conducted by Monte-Carlo re-sampling of the baseline with 1000 individuals using the Rannala and Mountain simulation option [23] in GeneClass2.

In addition to Bayesian cluster analysis as implemented in the program Structure, and genetic assignment as implemented in the program GeneClass2, the program NEWHYBRIDS v.1.1 [24] was used to assist identification of the suspected hybrid mother, her fetus, and the previously identified hybrid whale from 2007. This program is specifically designed to permit identification of species hybrids and back-cross categories between two potential donor species. This analysis was conducted once the potential contributory species and sub-species had been identified using the other statistical approaches described above. The program NEWHYBRIDS permits the posterior probability that each individual belongs to each of the distinct hybrid categories (e.g., F1 and back-cross variants) using the framework of Bayesian model-based clustering. Therefore, this program was specifically used to identify whether the suspected hybrids were F1 or back-crossed between the donor species. The program was run using default parameters.

## Results

### MtDNA analyses for the suspected hybrid and her fetus

The final data set (pregnant suspected hybrid whale, her female fetus, and minke whales worldwide examined in [13]) included the first 287 nucleotides of the mtDNA control region. The mtDNA sequences of the suspected hybrid mother whale and her fetus have been deposited in Genbank (accession numbers KC692324 and KC692325 respectively).

**Figure 2:**
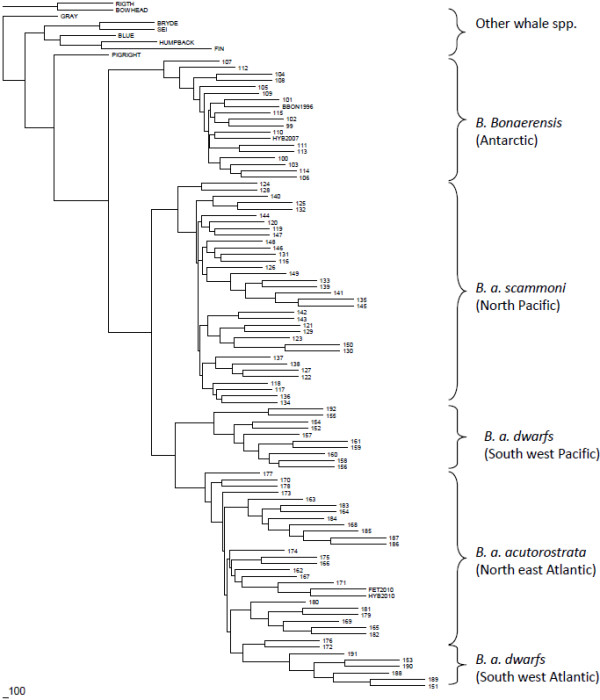
**Tree of the minke whale mtDNA haplotypes based on the neighbor-joining method.** Terminology for haplotypes is consistent with [4]. Sequence for the 2010 hybrid whale is indicated as ‘HYB2010’ while the fetus of this whale as ‘FET2010’ (identical haplotype). The 1996 Antarctic minke whale and the 2007 hybrid whale from [6] are denoted as BBON1996 and HYB2007, respectively.

The Kimura two-parameter distance between the suspected hybrid (or fetus), and the sequences of *B. bonaerensis* in [13] was estimated at 0.0715. In the comparison with *B. a. scammoni*, dwarf and *B. a. acutorostrata*, these estimates were 0.0224, 0.0202 and 0.0044, respectively. In the neighbour-joining based genealogy, the sequence of the suspected hybrid whale (and fetus) clustered within the *B. a. acutorostrata* clade (Figure 2). This demonstrates that this suspected hybrid has maternal contribution from *B. a. acutorostrata*.

### Microsatellite summary statistics for the species and sub-species

With the exception of the 9 dwarfs and the two potential hybrid whales genotyped here (mother and fetus), population genetic summary statistics (i.e., HWE, LD, allelic diversity, Ho, He etc.), and genotyping quality for the minke whale species and sub-species samples used in the present investigation have been previously documented [6]. Thus, some of the genetic data for these three species and sub-species are not repeated here. Given that the dwarfs were only represented by 9 individuals, the ability to compute reliable summary statistics for this sub-species is limited. Nevertheless, some of these statistics, including pair-wise F_ST_ values among the four species and sub-species have been computed (Table 2). These parameters provide a tentative estimation of the level of genetic similarity between the minke whale species and sub-species.

**Table 2:** **Genetic variation within (Allelic variation) and among (F**_**ST **_**values) the species and sub-species of minke whales based upon the analysis of 11 microsatellite loci**

**Species**	**N**	**Locus**											**Loci pooled**
		**1**	**2**	**3**	**4**	**5**	**6**	**7**	**8**	**9**	**10**	**11**	**A**_**T**_
Allelic variation													
Atl	91	2	2*	3	12	7	12	9	9	11	12	8	87
Pac	95	5	3	5	19	11*	15	13	9	12	8	13*	113
Ant	91	4	16	17	11	20**	37**	16	18**	39	47	16	241
Dwarf	9	3	1	3	8	3	8	3	4	9	7*	5	54
Total	286	9	16	20	28	22	39	19	18	47	52	16	286
F_ST_ values													
Atl x Pac		0.038	0.029	0.404	0.099	0.325	0.091	0.045	0.057	0.009	0.086	0.105	0.128
Atl x Ant		0.640	0.248	0.600	0.171	0.152	0.073	0.027	0.068	0.087	0.073	0.130	0.211
Pac x Ant		0.608	0.176	0.430	0.151	0.137	0.050	0.023	0.046	0.062	0.129	0.019	0.171
Dwarf x Pac		−0.016	0.184	0.377	0.045	0.178	0.036	0.313	0.109	0.023	0.078	0.076	0.144
Dwarf x Ant		0.615	0.293	0.502	0.130	0.250	0.031	0.308	0.095	0.042	0.062	0.087	0.225
Dwarf x Atl		0.003	0.097	0.616	0.106	0.451	0.075	0.360	0.043	0.029	0.069	0.209	0.216
Global F_ST_		0.529	0.184	0.481	0.137	0.227	0.070	0.067	0.060	0.052	0.094	0.089	0.0175
Global (P value)		<0.0001	<0.0001	<0.0001	<0.0001	<0.0001	<0.0001	<0.0001	<0.0001	<0.0001	<0.0001	<0.0001	<0.0001

Locus 1 = *DIrFCB14*, 2 = *EV104Mn*, 3 = *EV94Mn*, 4 = *EV001Pm*, 5 = *EV037Mn*, 6 = *GT509*, 7 = *GT211*, 8 = *GT575*, 9 = *GATA028*, 10 = *GATA417*, 11 = *GT023*. * = significant from HWE at 0.05, ** = significant deviation from HWE at 0.001. Atl = *B. a. acutorostrata*, Pac = *B. a. scammoni*, Ant = *B. bonaerensis*, Dwarf = *B. bonaerensis* unnamed sub species. A_T_ = total number of alleles. Note, Dwarf sample is based upon 9 individuals only, and therefore to be treated with caution.

Pair-wise F_ST_ estimates revealed that the four species and sub-species were genetically distinct to each other (Table 2). This also includes the dwarfs which have not been previously compared to the other three species and sub-species using this class of markers. That these species and sub-species are genetically distinct using these markers is important in order to be able to provide an unambiguous identification of the suspected hybrid and her fetus. Notably, several of the markers gave very high and in some pair-wise cases diagnostic (i.e., not overlapping allelic distributions) identification capacity among the species and sub-species. For example, the locus *DIrFCB14* gave a pair-wise F_ST_ of 0.64 between *B. a. acutorostrata* and *B. bonaerensis*, and several other markers gave similar levels of differentiation (Table 2).

### Genotype break-down for the suspected hybrid and her fetus

Comparison of the allelic profiles of the suspected hybrid whale and her fetus against the allelic profiles for the four species and sub-species demonstrated that neither of these individuals were pure (Table 3). One of the markers on its own, *DIrFCB14*, excluded the possibility of these individuals being pure species. When all possible combinations were examined, the genotypes for the mother and fetus could only be explained by being a hybrid between *B. a. acutorostrata* and *B. bonaerensis*. All other combinations of pure species and F1 hybrids were excluded by this manual inspection of the genotypic data. Thus, already based upon the mtDNA haplotype, and dissection of the genotypes for the suspected hybrid and her fetus, these data strongly indicate that she was an inter-species hybrid with maternal contribution from *B. a. acutorostrata*.

**Table 3:** Genotype compatibility for the suspected hybrid whale and her fetus with the allelic profiles for the four potential minke whale species and sub-species

**Marker**	**Mother**					**Fetus**				
	**Genotype**	***B.a.a***	***B.a.s***	***B.b***	**Dwarf**	**Genotype**	***B.a.a***	***B.a.s***	***B.b***	**Dwarf**
*DIrFCB14*	262	x	x		x	258	x	X		x
	266			x		266			x	
*EV104*	137			x		147	x	X	x	x
	147	x	x	x	x	147	x	X	x	x
*EV94*	195			x		195			x	
	211	x	x	x		213	x	X	x	x
*EV001*	134		x	x		153	x	X		
	153	x	x			157	x	X		
*EV37*	203	x	x	x		203	x	X	x	
	207	x		x		207	x		x	
*GT211*	102	x	x	x		102	x	x	x	
	110	x	x	x		108	x	x	x	
*GT509*	193	x		x		193	x		x	
	205	x	x	x	x	193	x		x	
*GT575*	146			x		154	x	x	x	x
	158	x	x	x		158	x	x	x	
*GATA028*	223		x	x		223		x	x	
	223		x	x		223		x	x	
*GATA417*	213	x	x	x	x	217	x	x	x	x
	229			x		229			x	
*GTO23*	105	x	x	x	x	103	x	x	x	
	117		x	x		105	x	x	x	x

X = allele present in that species or sub-species genetic baseline sample. Hybrid mother and her fetus’ genotypes presented as microsatellite allele size as calibrated in the NMDR [10]*. B.a.a* = *B.a. acutorostrata*, *B.a.s* = *B. a. scammoni*, *B.a* = *B. bonaerensis* dwarfs = *B. a. unnamed sub-species*. Note that the dwarfs were only represented by 9 individuals and therefore the absence of a specific allele in this species cannot be automatically be regarded as definitive evidence of this allele not existing for this sub-species.

### Statistical identification of paternity for the hybrid and her fetus

**Figure 3:**
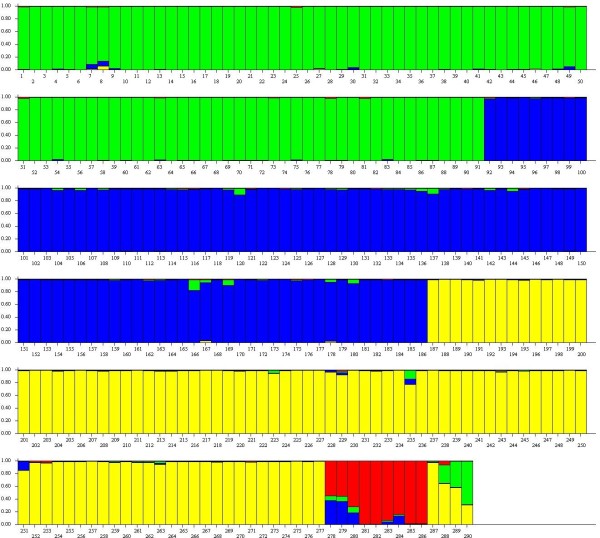
**Identification of the hybrid minke whale and her female fetus based upon Bayesian cluster analysis.** Each vertical line represents a single individual (which can be admixed), and each colour a genetic cluster. Columns 1–91 = *B. a. acutorostrata*, 92–186 = *B. a. scammoni*, 187–277 = *B. bonaerensis*, 278–286 = *B. a.* unnamed subspecies = “Dwarfs”, 287 = *B. bonaerensis* long-distance captured in the Arctic in 1996 [6], 288 = first documented hybrid between minke whale species captured also in the Arctic in 2007 [6], 289 = mother hybrid minke whale captured in 2010 documenting first pregnant hybrid between minke whale species, 290 = fetus for individual 289 representing the first documented example of back-crossing between any whale species.

Bayesian cluster analysis included all samples from the four species and sub-species. These analyses revealed several trends. First, supporting the results based upon F_ST_ (Table 2), large genetic differences were revealed among all of the minke whale species and sub-species (including the dwarfs) (Figure 3). Increasing the number of genetic clusters beyond 4 did not elucidate relationships among the species further, only revealing increased sub-structure within *B. bonaerensis* (Additional file 2: Figure S1). This trend probably reflects the fact that minke whales species in general may display some underlying or cryptic population genetic structure which was detected by Structure. Therefore, the results of cluster analysis are presented at *K* = 4 because it captures the interspecies relationships, and the ability to identify the hybrid and fetus (Figure 3). Second, both the hybrid and her fetus clearly displayed strong admixture between *B. a. acutorostrata* and *B. bonaerensis*. This was consistent both between iterative runs (data not presented) and also at different numbers of genetic clusters (Additional file 2: Figure S1). At *K* = 4, the hybrids’ estimated genotypic admixture proportions were 41% *B.a. acutorostrata* and 58% *B. bonaerensis*, whereas her fetus was estimated admixed 68% *B. a. acutorostrata* and 31% *B. bonaerensis* (Figure 3)*.*

The program NEWHYBRIDS [24] was used to estimate the posterior probabilities for the mother hybrid whale and her fetus for the categories of pure species, F1 hybrid, or back-cross to either species. The estimated probabilities using the default parameters were 0.94 for a F1 hybrid between *B. a. acutorostrata* and *B. bonaerensis* for the mother, and 0.99 for a back-cross to *B. a. acutorostrata* for the fetus. Thus, this analysis was consistent with all previous statistics presented above. These analyses strongly suggest that the fetus was sired by *B. a. acutorostrata*, demonstrating back-crossing into this species.

When using a genetic baseline as described in the materials and methods (i.e., all species and combinations of F1 hybrids without including the dwarfs), self-assignment simulations revealed highly accurate assignment among the species, sub-species and F1 hybrids simulated among them (93% correct self-assignment). This demonstrates powerful ability to conduct genetic assignment of the hybrid and her fetus, and is the result of the large genetic differences observed among these species and sub-species (Table 2) [6]. Assignment of the genetic profiles for the hybrid and her fetus were very similar (Table 4), and strongly concur with both results from Bayesian cluster analysis (Figure 3) and genotype scrutiny (Table 3). In short, it was possible to exclude the possibility that these two individuals were pure species, and furthermore, demonstrating that both individuals were hybrids between *B. a. acutorostrata* and *B. bonaerensis*.

**Table 4:** Identification of the hybrid whale and her fetus captured in the Northeast Atlantic in 2010

**Individual**	**Loci**	**Probability of false exclusion from baseline sample**						**Direct assignment**
		**Atl**	**Pac**	**Ant**	**Atl × Ant**	**Pac × Ant**	**Atl × Pac**	
Mother	11	<0.001	<0.001	<0.001	0.48	0.088	<0.001	Atl X Ant >99%
	8	<0.001	<0.001	<0.001	0.47	0.13	<0.001	Atl X Ant >99%
Fetus	11	<0.001	<0.001	<0.001	0.93	0.003	<0.001	Atl X Ant = 100%
	8	<0.001	<0.001	<0.001	0.83	<0.001	<0.001	Atl X Ant >99%

Exclusion (probability) and direct assignment (closest match) presented as implemented in Geneclass v. 2.0.

Atl = *B. a. acutorostrata*, Pac = *B. a. scammoni*, Ant = *B. bonaerensis*, Atl x Ant and Pac x Ant = simulated F1 hybrids.

### Clarification of paternity for the 2007 hybrid

The dwarf samples analysed here were not available for an earlier study which documented the first hybrid between minke whale species [6]. Thus, although mtDNA demonstrated maternal contribution was *B. bonaerensis*, paternity could not be unequivocally resolved for that hybrid. Within that study, examination of the genotype for the 2007 hybrid revealed two abonormalities. First, at the locus *GATA028*, the shorter allele (207 base pairs) displayed weaker PCR amplification than the longer allele (231 base pairs). This rare phenomenon was consistent between multiple DNA extractions and PCR runs. The second abnormality was that the hybrid displayed a 212 bp allele for the locus *GATA417*, and that this allele was not observed for any of the three species and sub-species included in that previous study (i.e., it was not observed in the approximately 100 individuals of *B. a. acutorostrata*, *B. a. scammoni* nor *B. bonaerensis*). Inspection of data for *B. a. acutorostrata* in the NMDR [10] showed that this 212 bp allele for *GATA417* was only observed in one other individual in the period 2007–2011, which included approximately 2500 individuals. Thus, this is a very rare allele observed for *B. a. acutorostrata*. In the present study however, which included microsatellite data for the dwarfs for the first time, this 212 bp allele for *GATA417* was observed as a homozygote in one of the 9 dwarf samples genotyped (Additional file 1: Table S1).

The Structure analyses gave an estimation of admixture components for the 2007 hybrid of 65% *B. bonaerensis*, 29% *B. a. acutorostrata,* and 6% dwarfs (Figure 3). The 6% clustering to the dwarfs in this program was entirely caused by the 212 bp allele at *GATA417* (i.e., when this locus was removed, genome allocation to the dwarfs was 0%). Analyses using the program NEWHYBRIDS, when including samples from *B. a. acutorostrata* and *B. bonaerensis*, estimated the posterior probability for this whale to be 0.97 back-cross to *B. bonaerensis*. When samples from dwarfs and *B. bonaerensis* were used in this analysis, the posterior probability was estimated as 0.99 back-cross to *B. bonaerensis*. However, when this individual whale’s genotype was changed from 231 231 at *GATA028* (which is the Norwegian database entry despite the fact that a 207 allele was consistently but weakly amplified) to 207 231, the posterior probabilities changed to 0.91 for a F1 hybrid between *B. a. acutorostrata* and *B. bonaerensis*, but remained strongly in favor of a back-cross to *B. bonaerensis* when samples from both dwarfs and *B. bonaerensis* were used in this analysis (probability 0.98). Thus, while the statistical analyses presented previously [6], and here, demonstrate this whale to be a hybrid between *B. bonaerensis* and *B. acutorostrata*, the sub-species from which the paternal contribution arises, and, whether this hybrid is a F1 or a back-cross remains unresolved.

## Discussion

While the level of speciation and degree of genetic exchange among minke whale species is not fully understood, based upon the analysis of mtDNA, it has been estimated that minke whales may have separated into two main species approximately 5 million years ago, and further into allopatric sub-species approximately 1.5 million years ago [4]. The analyses presented here, include for the first time, analysis of all four species and sub-species using microsatellite markers. While these data are limited by the low number of dwarfs, all four species and sub-species were demonstrated to be genetically distinct with this class of markers, supporting previous analyses using mtDNA [3,4]. The data set was thereafter used to identify a whale captured in the North east Atlantic in 2010, that was demonstrated to be a hybrid between *B. bonaerensis* and *B. acutostrata*. This hybrid’s paternity was demonstrated to be *B. a. acutorostrata*, and significantly, she was pregnant. Therefore, these data demonstrate the potential fertility of hybrids between minke whales, and the potential for back-crossing among these species.

### Identification of the hybrids

In the first observation of hybridisation between minke whale species [6], paternal contribution to the hybrid captured north of the Arctic circle in 2007 was not conclusively resolved. That specific hybrid’s mother was demonstrated to be *B. bonaerensis* based upon her mtDNA profile, and the paternal contribution most likely to be *B. a. acutorostrata*. The dwarfs were not available for that study, and as such it was not possible to unequivocally resolve paternity. The extra analyses for this individual presented here have shed more light on the identification of that 2007 hybrid. However, even after the inclusion of genetic data from dwarfs, it is still not possible to conclusively resolve paternity for this hybrid. In part, this challenge is due to the low number of dwarf samples available for analysis, in addition to the low number of diagnostic markers.

In the present study, a second hybrid, captured in the Arctic in 2010, was genetically identified. What is significant about this second hybrid is that she was pregnant, documenting that hybrids between these two species can be fertile, and potentially produce offspring. All analyses presented here were consistent and strongly indicated that she was sired by *B. a. acutorostrata*, and analysis of her female fetus strongly suggested that she in turn paired with a second *B. a. acutorostrata*, effectively demonstrating back-crossing between hybrids and this species. These data represent only the second documented example of fertility among interspecific *Balaenopteridae* whale hybrids in the wild. Furthermore, this result is of broad significance due to the fact that interspecific hybrids between mammals are more frequently infertile than in other vertebrates, a phenomena which is linked to the fact that hybrid inviability evolves faster in mammals than in other vertebrates for example birds [25,26].

### Mechanisms behind the hybridizations and implications

The first observed *B. bonaerensis* individual north of the Arctic circle was a male in 1996 [6]. Approximately a decade later (2007), the first documented hybrid between *B. acutorostrata* and *B. bonaerensis* was reported [6]. This female whale displayed maternal contribution from *B. bonaerensis*, but as described above, the paternal contribution could not be unequivocally resolved between *B. a. acutorostrata* and dwarfs*.* These observations nevertheless demonstrate that at least two whales with *B. bonarensis* mtDNA haplotypes have also migrated into the northern hemisphere which is beyond their previously documented geographic ranges. The latest documented hybrid, a pregnant female captured in 2010, displayed paternal contribution from *B. bonaerensis* which means that its mother was *B. a. acutorostrata*. Thus, it appears that hybridisation between these two species is occurring with both maternal and paternal contribution from *B. bonaerensis*.

Inter-oceanic migrations are rarely documented in whales, and have only been documented for the humpback (*Megaptera novaeangliae*) [27] and Antarctic minke whales [6]. As discussed previously [6], it is not possible to resolve whether the observed migrations of *B. bonaerensis* to the Arctic, and hybridisation between this and *B. a. acutorostrata* are rare random events that have occurred over a longer period of time, the result of a low number of *B. bonaerensis* migrating from the Antarctic to the Arctic in the 1990’s, or alternatively, represent a trend that is increasing in frequency. The NMDR for *B. a. acutorostrata* only goes back as far as 1996 [10], and as such it is not possible to exclude any of these possibilities. Furthermore, while hybrids between minke whale species have not been observed among the >6000 minke whales contained in the Japanese DNA register for minke whales captured in the Antarctic (N. Kanda pers. obs.), it is only based upon 6 microsatellites, and only one of which is partially diagnostic between these species. Therefore, the potential presence of minke whale hybrids in the southern hemisphere cannot be excluded at the present. A long-term monitoring of both the Japanese DNA register and the NMDR will be required in order to elucidate this situation.

Interspecific hybridisation in the wild has been documented in a range of ecosystems and taxa, also in mammals. While this may occur naturally, the frequency of interspecific hybridization appears to be increasing in general as a result of anthropogenic influence [28,29]. This can in turn challenge existence of species [28,30] and cause a range of complicated management challenges [31]. Documentation of fertility among interspecific mammalian hybrids in the wild are relatively rare, and specifically for *Balaenopteridae* whales, have only been previously reported for hybrids between fin and blue whales [8,9]. Of the hybrids detected between fin and blue whales, it appears that not all are fertile [9]. Whether or not the pregnant hybrid minke whale observed in the present study represents the normal result of hybridization between these two species or not, and in turn, whether her fetus would have been born alive, and been reproductively viable herself, remains to be seen.

The analyses presented here demonstrate the potential for reproductive compatibility between *B. acutorostrata* and *B. bonaerensis*. Nevertheless, unless the frequency of reproductive contact increases significantly in the future, it is unlikely that the boundary between these two species will be challenged. Reproductive incompatibility of hybrids represents a significant process by which hybrid zones between two species do not lead to break down of the pure species [e.g., [32]. However, there are also other biological mechanisms which limit the degree to which two genetically compatible species overlapping in time and space hybridise. For example, the three species of fur seals inhabiting Macquarie island south of Australia and New Zealand have displayed significant hybridisation and can back-cross [33]. Nevertheless, the frequency of interspecific hybridsation on this Island appears to have decreased in recent years, and behavioral mechanisms [34], as well as partially impaired reproductive characteristics for hybrid males [32] has limited the hybridisation between these species.

### Further work

The ability to detect hybrids with molecular genetics tools is heavily dependent upon the species/populations displaying clear genetic differences to each other in a range of genetic markers [e.g., [35]. Once hybrids start to reproduce and back-cross, the ability to differentiate among F2, F3 and back-crossed individuals becomes increasingly challenging [33]. Greater numbers of species-informative nuclear loci (i.e., 50+) are required to provide statistical resolution if individuals are to be accurately divided into F2, F3 and multiply back-crossed variants [36,37]. The NMDR contains genetic profiles for >8000 minke whales captured in the Northeast Atlantic in the period 1996-present [10]. While the microsatellite markers implemented in the present study provide powerful ability to almost diagnostically resolve F1 hybrids between all of the minke whale species and sub-species, it is not possible to exclude the possibility that other back-crossed or multiply back-crossed individuals exist within the NMDR. Nevertheless, mtDNA is maternally inherited and does not display recombination. Thus, the fact that only two individuals within the NMDR have displayed mtDNA sequences deviating from *B. a. acutorostrata* may suggest that at least back-crossed whales with maternal *B. bonaerensis* contribution are very rare in the North east Atlantic. Nevertheless, elucidating the frequency of potential back-crossing between these two species where the mother is *B. a. acutorostrata* and father is *B. bonaerensis* will be important in order to elucidate what evolutionary mechanisms are involved in these recent observations. In order to fully elucidate the level of genetic contact between minke whale species and sub-species, the development of large resources of species informative single nucleotide polymorphisms distributed throughout the genome will be required.

## Conclusion

This study clearly demonstrates, for the first time, that hybrids between minke whale species may be fertile, and that they can back-cross. Whether contact between these species represents a contemporary event linked with documented recent changes in the Antarctic ecosystem, or has occurred at a low frequency over many years, remains open.

## Authors’ contributions

KAG conceived the study, performed data quality checks, conducted statistical analysis with the microsatellite data, and wrote the first draft of the manuscript. NK genotyped the “dwarfs” and calibrated their microsatellite scores between the two DNA registers. TH provided biological information about the mother and her fetus. LAP conducted statistical analysis with mtDNA data and produced Figure 2. NØ conceived the biological hypotheses underlying these results. BBS and AGES conducted all microsatellite genotyping, mtDNA sequencing, and preliminary data quality checks. HJS assisted in biological and statistical interpretation, and ran the program NEWHYBRIDS. All authors contributed to the overall design of the study, interpretation and presentation of the results, and approved the final manuscript. KAG and TH are jointly responsible for sample storage and genotyping samples in the Norwegian minke whale DNA register which is owned by the Norwegian Directorate of Fisheries. All authors read and approved the final manuscript.

## Supplementary Material

Additional file 1: Table S1 Calibration of the microsatellite DNA data between the 9 “dwarfs” and the rest of the samples analysed in the present study. Click here for file

Additional file 2: Figure S1 Bayesian clustering analysis computed for the entire data set when the number of genetic clusters is set at 2 (top figure) through to 6 (bottom figure). Click here for file
