# Supplementary material for: Hybrids between common and Antarctic minke whales are fertile and can back-cross
Source: BMC Genet. 2013 Apr 15;14:25. doi: 10.1186/1471-2156-14-25 (PMC3637290; doi:10.1186/1471-2156-14-25)
Supplement: Additional file 2: Figure S1 — Bayesian clustering analysis computed for the entire data set when the number of genetic clusters is set at 2 (top figure) through to 6 (bottom figure). [file 1471-2156-14-25-S2.doc]

Supplementary Figure 1. Glover et al. 2013. Hybrids between common and Antarctic minke whales are fertile and can back-cross.

Bayesian clustering analysis computed when the number of genetic clusters is set at 2 (top figure) through to 6 (bottom figure). Each vertical line represents a single individual (which can be admixed), and each colour a genetic cluster. Columns 1-91 = *B. a. acutorostrata*, 92-186 = *B. a. scammoni*, 187-277 = *B. bonaerensis*, 278-286 = *B. a.* unnamed subspecies = “Dwarfs”, 287 = *B. bonaerensis* long-distance captured in the Arctic in 1996 , 288 = first documented hybrid between minke whale species captured also in the Arctic in 2007 , 289 = mother hybrid minke whale captured in 2010 documenting first pregnant hybrid between minke whale species, 290 = fetus for individual 289 representing the first documented example of back-crossing between any whale species.

K2

K3

K4

K5

K6

1. Glover KA, Kanda N, Haug T, Pastene LA, Øien N, Goto M, Seliussen BB, Skaug HJ: **Migration of Antarctic Minke Whales to the Arctic**. *Plos One* 2010, **5**(12).
